# Regional variation in chronic kidney disease and associated factors in hypertensive individuals in rural South Asia: findings from control of blood pressure and risk attenuation—Bangladesh, Pakistan and Sri Lanka

**DOI:** 10.1093/ndt/gfy184

**Published:** 2018-07-05

**Authors:** Liang Feng, Hithanadura Asita de Silva, Imtiaz Jehan, Aliya Naheed, Anuradhani Kasturiratne, Gulshan Himani, Mohammad Abul Hasnat, Tazeen H Jafar

**Affiliations:** 1 Program in Health Services & Systems Research, Duke-NUS Medical School, Singapore; 2 Clinical Trials Unit, Department of Pharmacology, Faculty of Medicine, University of Kelaniya, Kelaniya, Sri Lanka; 3 Department of Community Health Science, Aga Khan University, Karachi, Pakistan; 4 Initiative for Noncommunicable Diseases, Health Systems and Population Studies Division, icddr,b, Dhaka, Bangladesh; 5 Department of Public Health, Faculty of Medicine, University of Kelaniya, Kelaniya, Sri Lanka; 6 Department of Family Medicine, Aga Khan University, Karachi, Pakistan; 7 Kurmitola General Hospital, Dhaka, Bangladesh; 8 Department of Renal Medicine, Singapore General Hospital, Singapore; 9 Duke Global Health Institute, Duke University, Durham, NC, USA

**Keywords:** chronic kidney disease, hypertension, prevalence, risk factors, South Asia

## Abstract

**Background:**

We aimed to determine the prevalence of chronic kidney disease (CKD) and its cross-country variation among hypertensive individuals in rural Bangladesh, Pakistan and Sri Lanka. We also explored the factors associated with CKD in these populations.

**Method:**

We studied baseline data from the Control of Blood Pressure and Risk Attenuation-Bangladesh, Pakistan and Sri Lanka (COBRA-BPS) trial, an ongoing cluster randomized controlled trial on 2643 hypertensive adults ≥40 years of age from 30 randomly selected rural clusters, 10 in each of the three countries. CKD was defined as an estimated glomerular filtration rate (eGFR) <60 mL/min/1.73 m^2^ or a urine albumin:creatinine ratio (UACR)  ≥30 mg/g. Determinants for CKD were assessed using logistic regression analysis.

**Results:**

The overall prevalence of CKD was 38.1% (95% confidence interval 36.2–40.1%): 21.5% with eGFR <60 mL/min/1.73 m^2^ and 24.4% with UACR ≥30 mg/g. CKD prevalence varied across the three countries (58.3% in Sri Lanka, 36.4% Bangladesh and 16.9% Pakistan; P <0.001). The factors independently associated with higher odds of CKD were older age, being unmarried, higher 24-h urinary sodium excretion, presence of diabetes, elevated systolic blood pressure, diuretic use and living in Bangladesh or Sri Lanka (versus Pakistan).

**Conclusions:**

The prevalence of CKD is alarmingly high in community-dwelling hypertensive adults, with significant cross-country variation in South Asia. Our findings underscore the urgency for further research into the etiology of CKD and address associated factors in targeted public health strategies with hypertension care outreach services in rural South Asia.

**ClinicalTrials.gov:**

NCT02657746

## INTRODUCTION

Chronic kidney disease (CKD), defined as a reduced estimated glomerular filtration rate (eGFR) or the presence of albuminuria is associated with progression to end-stage renal disease (ESRD) and increased risks of premature mortality from cardiovascular disease (CVD) [1–3]. CKD ranked as one of the most rapidly increasing causes of mortality globally [4], with a greater burden in low- and middle-income countries (LMICs) than high-income countries [5, 6].

The prevalence of CKD reported primarily from urban parts of South Asia has ranged from 7.2% to 17.2% in the general population [7–11]. Most of South Asia is still rural (65% Bangladesh, 61% Pakistan, 67% India, 82% Sri Lanka) and health care infrastructure and provider characteristics are very different from the urban setting and CVD case fatality rates are higher than in urban areas [12, 13]. Previously we demonstrated that simple strategies such as physician education on hypertension management coupled with home health education on a healthy lifestyle are effective in lowering blood pressure (BP) and preserving kidney function in individuals with hypertension in Pakistan [14, 15]. However, information on the prevalence and risk factors of CKD is scarce among hypertensive individuals from rural areas in Pakistan and regional South Asian countries. Moreover, existing literature from the region relies on studies using different protocols and variable definitions, adding challenges to interpretation for any comparison on the prevalence of CKD in different countries within a region and related preventive efforts.

To fill this gap, we analyzed cross-sectional data collected during the baseline screening of the ongoing cluster randomized controlled trial of representative community-dwelling hypertensive individuals in Bangladesh, Pakistan and Sri Lanka with the following objectives: (i) to determine the prevalence and cross-country variation in CKD among individuals with hypertension in rural communities in Bangladesh, Pakistan and Sri Lanka and (ii) to evaluate whether the sociodemographic factors, lifestyle and comorbid conditions are associated with CKD in these populations and whether they account for potential cross-country variation in CKD. We hypothesized that the prevalence of CKD will be high, and will vary among hypertensive individuals in rural communities across the three South Asian countries, and (ii) the cross-country variation in CKD cannot be fully accounted for by differences in sociodemographics, lifestyle factors or comorbid conditions.

## MATERIALS AND METHODS

### Population

The present study was performed using baseline data from the Control of Blood Pressure and Risk Attenuation-Bangladesh, Pakistan and Sri Lanka (COBRA-BPS) full-scale study. Detailed information on the study is provided elsewhere [16]. Briefly, the COBRA-BPS full-scale study is an ongoing 2-year randomized controlled trial among 2643 hypertensive adults from 30 randomly selected rural clusters (communities), 10 clusters each, in Bangladesh, Pakistan and Sri Lanka. In each country, cluster selection was stratified by the distance (≤2.5 km for near and >2.5 for far) from government primary care clinics such that there were six near and four far clusters in each country. Individuals in each cluster were screened using a door-to-door sampling method. The inclusion criteria for COBRA-BPS were age ≥40 years, hypertension [defined as sustained elevation of systolic blood pressure (SBP) to ≥140 mmHg or diastolic blood pressure (DBP) to ≥90 mmHg based on two readings from two separate days, or receiving antihypertensive medications] and a resident in the selected cluster. Individuals were excluded if they had severe physical incapacity, were pregnant, had advanced diseases (on dialysis, liver failure and other systemic diseases) or were mentally compromised leading to inability of giving consent.

A total of 11 510 individuals ≥40 years of age were screened for hypertension to be recruited in the COBRA-BPS trial in the 30 clusters in rural areas of the three countries. As shown in Supplementary data, Figure S1, of the 11 510 individuals, 2977 (25.9%) had hypertension, 7878 (68.4%) were normotensive and 655 (5.7%) had undetermined BP status. The proportion of individuals with hypertension was 36.5% in Bangladesh, 21.6% in Pakistan and 39.8% in Sri Lanka. Of the 2977 hypertensive individuals in three countries, 334 were excluded for various reasons (Supplementary data, Figure S1). Thus 2643 hypertensive individuals were enrolled in the trial from 2240 households, with 895 individuals enrolled from 777 households in Bangladesh, 894 from 736 in Pakistan and 854 from 727 in Sri Lanka. The number of participants per cluster ranged from 84 to 102, with an average of 88 per cluster. Of the 2643 hypertensives recruited, 294 (11.1%) were excluded due to missing laboratory data on serum creatinine (*n* = 248) or urine albumin (*n* = 278), leaving 2349 individuals for the analysis of CKD (Supplementary data, Figure S1). The comparison of characteristics of individuals with hypertension included (*n* = 2349) and excluded (*n* = 294) from the analysis of CKD is shown in Supplementary data, Table S1.

The study protocol was approved by the Institutional Review Board of the National University of Singapore, Singapore; Ethical Review Committee of the International Centre for Diarrhoeal Disease Research, Bangladesh; the Ethical Review Committee of Aga Khan University, Pakistan; the Ethics Review Committee of the Faculty of Medicine, University of Kelaniya, Sri Lanka and the Interventions Research Ethics Committee of the London School of Hygiene and Tropical Medicine, London, UK. All study participants provided written informed consent.

### Measurements

Self-reported sociodemographics (age, gender, education, marital status, employment status and economic status), lifestyle characteristics (smoking, physical activity and frequency of fruits and vegetables consumption), medical history (self-reported heart disease, stroke and CKD) and current medication use were obtained at the baseline visit. The economic status was determined by the International Wealth Index (IWI) [17]. Physical activity was evaluated by the short version of the International Physical Activity Questionnaire (IPAQ), with good reliability and validity [18]. Diabetes was defined as a fasting plasma glucose ≥126 mg/dL or self-reported use of anti-diabetes medication. CVD was defined as self-reported heart disease or stroke.

On enrolment, the participant’s weight, height, waist circumference and BP were measured. Body mass index (BMI) was calculated as weight (in kg)/height (in m^2^). BP was measured four times every 5 min of rest in a sitting position using an HEM-7300 digital monitor (Omron, Kyoto, Japan). The mean of the last two readings was used in the analysis.

An overnight fasting blood sample was collected to measure serum creatinine (measured on a Beckman DU; Beckman Coulter, Brea, CA, USA), lipids (measured on a Hitachi 912; Roche, Basel, Switzerland) and plasma glucose (measured on Synchron Cx-7/Delta; Beckman Coulter) in a local laboratory in each country. Serum creatinine measurements were calibrated to isotope dilution mass spectrometry (IDMS)-traceable values. Glomerular filtration rate was estimated using the original Chronic Kidney Disease Epidemiology Collaboration (CKD-EPI) equation [19].

Urinary albumin, creatinine and sodium were measured from a spot urine sample in the same local laboratory in each country. Urine albumin and creatinine excretion were measured by nephelometry using the array systems method. Urine sodium was determined with a Synchron Cx-7 analyser using an enzymatic colorimetric assay. All tests were performed to a standard protocol that conformed to the international standards for definitions and measurements. Albuminuria was evaluated by the urine albumin:creatinine ratio (UACR).

The 24-h urinary excretion of sodium was estimated using the Kawasaki *et al.* [20], Tanaka *et al.* [21], and Mage *et al.* formulas [22, 23].

### Analysis methods

The primary outcome of interest in the current study was CKD, defined as an eGFR <60 mL/min/1.73 m^2^ or UACR ≥30 mg/g based on the Kidney Disease: Improving Global Outcomes (KDIGO) definition [24].

Among those with eGFR <60 mL/min/1.73 m^2^, the following KDIGO classification was applied: G3a, eGFR 45–59 mL/min/1.73 m^2^; G3b, eGFR 30–44 mL/min/1.73 m^2^; G4, eGFR 15–29 mL/min/1.73 m^2^ and G5, eGFR <15 mL/min/1.73 m^2^ [24]. Albuminuria was categorized into three groups: A1, UACR <30 mg/g; A2, UACR 30–300 mg/g and A3, UACR >300 mg/g [24].

The characteristics of hypertensive individuals were expressed using count and percentage for categorical variables and mean and standard deviation (SD) or median and interquartile range (IQR), when appropriate, for continuous variables. Comparison between individuals across three countries was performed using a one-way analysis of variance for continuous variables and the chi-square test for categorical variables. When continuous variables were not normally distributed, the Kruskal–Wallis test was used. Age- and gender-standardized prevalence of CKD for each country were estimated by a direct method using the combined sample of the three countries as the standard population. We used logistic regression to investigate the possible association between risk factors and CKD in our combined sample. Two models were constructed. In Model 1 we included sociodemographic characteristics [age, gender, education, marital status, wealth index score (classified by quintiles)], lifestyle behaviors (physical activities score, smoking, fruit consumption per week, 24-h urine sodium excretion) and country. In Model 2 we further adjusted for clinical and comorbid characteristics, including BMI, waist circumference, diabetes, SBP, DBP, CVD, high-density lipoprotein (HDL), low-density lipoprotein (LDL), triglyceride and use of antihypertensive agents [diuretics, angiotensin-converting enzyme inhibitor (ACEI), angiotensin II receptor blocker (ARB), calcium channel blocker (CCB) and β-blocker]. Because ∼20% households had two or more family members selected, we performed mixed-effects logistic regression using SAS ‘PROC GLIMMIX’ (SAS Institute, Cary, NC, USA) to account for clustering within the household and village. Both random household and random village effects were introduced in the models.

To assess whether the association with CKD varied across the three countries, we investigated two-way interactions between country and each variable in the model. When significant interactions were observed, we reported the ratio of odds ratios (RORs) [25], namely, the OR for one variable for one country (numerator country) divided by that for the same variable in another country. The ROR is the exponentiated regression coefficient of the interaction term between the variable and country. An ROR >1 with a P-value <0.05 indicates a stronger association between the variable and CKD in the numerator country than in the denominator country; when it is <1 and statistically significant, the association is weaker in the numerator country. We also performed subgroup analysis by country if significant interactions with variables on the outcome of CKD were detected.

Sensitivity analysis was done, where we reassessed the association of CKD by estimating GFR using the CKD-EPI Pakistan equation (a modified version of the CKD-EPI creatinine-based equation for Pakistanis) [26] and estimating 24-h urinary sodium excretion using Tanaka *et al.* [21] and Mage *et al.* formulas [22, 23]. All analyses were conducted using SAS version 9.3 (SAS Institute) and all hypothesis testing was two-tailed with P < 0.05 set as statistically significant.

## RESULTS

### Characteristics of participants

Supplementary data, Table S1 summarizes the baseline characteristics of the hypertensive individuals included (*n* = 2349) and excluded (*n* = 294) from the analysis of CKD. Compared with the individuals included, those excluded were more likely to be uneducated and unmarried. The latter also had lower IWI scores and physical activity; a lower prevalence of diabetes, CVD and antihypertensive and higher DBP.

The mean age of participants was 58.8 (SD 11.3) years, 64.5% (*n* = 1514) were female, 61% (*n* = 1426) had a formal education and 27% (*n* = 627) had diabetes (Table 1).

**Table 1:** Baseline characteristics of individuals with hypertension by countries (*n* = 2349)

Characteristics	Total (*N* = 2349)	Bangladesh (*n* = 868)	Pakistan (*n* = 685)	Sri Lanka (*n* = 796)	P-value
Sociodemographics					
Age (years), mean (SD)	58.8 (11.3)	56.7 (11.2)	56.7 (11.2)	62.9 (10.4)	<0.001
Male, *n* (%)	835 (35.6)	314 (36.2)	269 (39.3)	252 (31.7)	0.008
Education, *n* (%)					<0.001
Formal	1426 (60.7)	450 (51.8)	209 (30.5)	767 (96.4)	
Informal	923 (39.3)	418 (48.2)	476 (69.5)	29 (3.6)	
Marital status, *n* (%)					<0.001
Unmarried	620 (26.4)	181 (20.9)	173 (25.3)	266 (33.4)	
Married	1729 (73.6)	687 (79.1)	512 (74.7)	530 (66.6)	
Employment (yes), *n* (%)	673 (28.7)	230 (26.5)	234 (34.2)	209 (26.3)	<0.001
IWI, *n* (%)					<0.001
≥0–<39.8	468 (20.0%)	227 (26.1)	215 (31.7)	26 (3.3)	
≥39.8–<54.10	469 (20.0%)	258 (29.7)	129 (19.0)	82 (10.3)	
≥54.10–<65.66	468 (20.0%)	233 (26.8)	119 (17.5)	116 (14.6)	
≥65.66–<78.10	468 (20.0%)	101 (11.6)	144 (21.2)	223 (28.1)	
≥78.10–<100	469 (20.0%)	49 (5.7)	72 (10.6)	348 (43.8)	
Lifestyle behaviors					
Smoking, *n* (%)	242 (10.3)	105 (12.1)	95 (13.9)	42 (5.3)	<0.001
Physical activity score, MET (min/week), median (IQR)	3360 (693–6760)	3360 (1386–6720)	2309 (248–6852)	3066 (693–7116)	<0.001
≥1 fruit consumption per week, *n* (%)	1460 (62.3)	540 (62.2)	317 (46.3)	603 (76.1)	<0.001
≥1vegetable consumption per week, *n* (%)	2319 (98.9)	853 (98.3)	679 (99.1)	787 (99.4)	0.08
24-h urine sodium (mg/day), mean (SD)	4655.4 (1533.5)	4512.8 (1565.5)	4184.3 (1470.6)	5218.6 (1371.2)	<0.001
Clinical and comorbid factors					
BMI, mean (SD)	24.7 (5.0)	24.2 (4.0)	25.0 (6.2)	25.1(4.7)	<0.001
Waist circumference (cm), mean (SD)	88.5 (20.9)	86.1 (10.8)	90.0 (34.1)	89.8 (12.3)	<0.001
SBP (mmHg), mean (SD)	145.6 (21.7)	141.6 (20.7)	149.1 (21.1)	146.9 (22.7)	<0.001
DBP (mmHg), mean (SD)	88.3 (14.1)	87.1 (13.7)	91.5 (13.9)	86.7 (14.4)	<0.001
HDL (mg/dL), mean (SD)	45.2 (12.9)	38.2 (10.4)	42.4 (11.9)	55.3 (9.3)	<0.001
LDL (mg/dL), mean (SD)	124.6 (40.6)	133.4 (40.3)	108.7 (34.1)	128.7 (42.1)	<0.001
Triglyceride (mg/dL), median (IQR)	129.0 (94.0–183.0)	145.9 (105.4–207.9)	136.0 (98.0–194.0)	108.8 (85.1–143.7)	<0.001
eGFR (mL/min/1.73 m^2^), mean (SD)	83.6 (26.1)	88.1 (21.0)	105.6 (19.8)	59.8 (13.9)	<0.001
UACR (mg/g), median (IQR)	13.7 (5.9–29.5)	11.6 (4.1–39.3)	7.0 (4.0–17.1)	19.8 (13.1–32.0)	<0.001
Diabetes, *n* (%)	627 (26.7)	194 (22.4)	130 (19.0)	303 (38.1)	<0.001
CVD, *n* (%)	543 (23.7)	281 (34.1)	88 (12.9)	174 (22.1)	<0.001
Heart disease, *n* (%)	316 (13.8)	150 (18.3)	48 (7.1)	118 (15.0)	<0.001
Stroke, *n* (%)	301 (12.8)	179 (20.6)	51 (7.5)	71 (8.9)	<0.001
Any antihypertensive use, *n* (%)	1578 (67.2)	650 (74.9)	285 (41.6)	643 (80.8)	<0.001
ARB or ACEI use, *n* (%)	800 (34.1)	193 (22.2)	101 (14.7)	506 (63.6)	<0.001
CCB use, *n* (%)	508 (21.6)	243 (28.0)	57 (8.3)	208 (26.1)	<0.001
Diuretics use, n (%)	248 (10.6)	48 (5.5)	23 (3.4)	177 (22.2)	<0.001
β-blocker use, *n* (%)	620 (26.4)	396 (45.6)	140 (20.4)	84 (10.6)	<0.001

Number missing was 7 for IWI, 1 for smoking, 26 for physical activity score, 4 for frequency of fruit and vegetable consumption, 5 for 24-h sodium excretion, 5 for BMI, 1 for LDL and triglyceride, 54 for CVD and 61 for heart disease.

Mann–Whitney U test for physical activities score, triglyceride and UACR.

MET, metabolic equivalence of task.

### Characteristics of individuals by countries

Table 1 shows the characteristics of individuals by three countries. The distribution differed significantly across countries for all characteristics but the frequency of vegetable consumption (P < 0.05). Compared with individuals in Bangladesh and Pakistan, those in Sri Lanka were older, less likely to be male, better educated, more often unmarried, had higher socio economic status, higher 24-h urine sodium excretion, higher UACR, higher prevalence of diabetes and lower eGFR. Individuals from Bangladesh were more likely to be married, had lower socioeconomic status, lower SBP and higher prevalence of CVD than those in the other two countries. Individuals in Pakistan were more likely to be male, less educated, had lower 24-h urine sodium excretion, lower UACR, lower proportion of diabetes and CVD as well as higher SBP and eGFR than those in Bangladesh and Sri Lanka.

### Prevalence of CKD stratified by country

Table 2 shows the prevalence of CKD in different stages by countries. The overall prevalence of CKD was 38.1% (95% CI 36.2–40.1): the prevalence of eGFR <60 mL/min/1.73 m^2^ was 21.5% (95% CI 19.8–23.1) and the prevalence of UACR  ≥30 mg/g was 24.4% (95% CI 22.6–26.2). The crude prevalence of CKD in Sri Lanka was 58.3% (95% CI 54.8–61.8), ∼1.6 times that in Bangladesh [36.4% (95% CI 33.2–39.7)] and 3.4 times that in Pakistan [16.9% (95% CI 14.1–19.8)]. The cross-country variation in CKD persisted after standardization by age and gender. However, the prevalence of albuminuria in Bangladesh [29.6% (95% CI 26.5–32.7)] and Sri Lanka [(25.9% (95% CI 22.8–29.0)] was comparable, but both were much higher than in Pakistan [16.1% (95% CI 13.2–18.9)].

**Table 2:** **Overall and country-specific prevalence** ^a^ **of various stages of CKD among individuals with hypertension (*n* = 2349)**

Characteristics	Total (*N* = 2349)	Bangladesh (*n* = 868)	Pakistan (*n* = 685)	Sri Lanka (*n* = 796)
CKD stage G3, A2 or worse	896 [38.1 (36.2–40.1)]	316 [36.4 (33.2–39.7)]	116 [16.9 (14.1–19.8)]	464 [58.3(54.8–61.8)]
Age-standardized prevalence^b^, % (95% CI)	–	38.4 (34.1–42.8)	17.3 (14.1–20.5)	49.8 (45.0–54.5)
Age- and gender-standardized prevalence^b^, % (95% CI)	–	37.8 (33.5–42.1)	17.2 (14.0–20.3)	49.2 (44.4–53.9)
CKD G3 or worse only	504 [21.5 (19.8–23.1)]	102 [11.8 (9.6–14.0)]	20 [2.9 (1.6–4.3)]	382 [48.0 (44.5–51.5)]
CKD A2 or worse only	573 [24.4 (22.6–26.2)]	257 [29.6 (26.5–32.7)]	110 [16.1 (13.2–18.9)]	206 [25.9 (22.8–29.0)]
CKD G3a only	348 [14.8 (13.4–16.3)]	68 [7.8 (6.0–9.7)]	10 [1.5 (0.5–2.4)]	270 [33.9 (30.6–37.3)]
CKD G3b only	120 [5.1 (4.2–6.0)]	23 [2.7 (1.5–3.8)]	4 [0.6 (0.0–1.2)]	93 [11.7 (9.4–14.0)]
CKD G4-5 only	36 [1.5 (1.0–2.1)]	11 [1.3 (0.5–2.1)]	6 [0.9 (0.1–1.7)]	19 [2.4 (1.3–3.5)]
A1 only	1776 [75.6 (73.9–77.4)]	611 [70.4 (67.3–73.5)]	575 [83.9 (81.1–86.8)]	590 [74.1 (71.0–77.2)]
A2 only	495 [21.1 (19.4–22.7)]	215 [24.8 (21.8–27.7)]	93 [13.6 (10.9–16.2)]	187 [23.5 (20.5–26.5)]
A3 only	78 [3.3 (2.6–4.1)]	42 [4.8 (3.4–6.3)]	17 [2.5 (1.2–3.7)]	19 [2.4 (1.3–3.5)]

Values presented as *n* [% (95% CI)] unless otherwise noted. Stage G3, A2 or worse: eGFR <60 ml/min/1.73 m^2^ or UACR ≥30 mg/g; G3 or worse only: eGFR <60 mL/min/1.73 m^2^; A2 or worse only: UACR ≥30 mg/g; G1, eGFR ≥90 mL/min/1.73 m^2^; G2, eGFR ≥60 mL/min/1.73 m^2^–<90 mL/min/1.73 m^2^; G3, eGFR ≥30 mL/min/1.73 m^2^–< 60 mL/min/1.73 m^2^; G3a, eGFR ≥45 mL/min/1.73 m^2^–<60 mL/min/1.73 m^2^; G3b, eGFR ≥30 mL/min/1.73 m^2^–<45 mL/min/1.73 m^2^; G4, eGFR ≥15 mL/min/1.73 m^2^ –<30 mL/min/1.73 m^2^; G5, eGFR ≤15 mL/min/1.73 m^2^; A1, UACR <30 mg/g; A2, UACR ≥30 –≤300 mg/g; A3, UACR >300 mg/g.

a Crude prevalence was reported unless otherwise indicated.

b Total sample (*n* = 2349) was used as the standard population.

Supplementary data, Tables S2–5 provide age- and gender-specific prevalence of CKD Stages 3–5 by country.

### Factors associated with CKD

The factors associated with CKD in all three countries are shown in Table 3. In the analysis in Model 1 adjusted for sociodemographic variables, older age [OR 1.06 (95% CI 1.05–1.07)], being unmarried [OR 1.45 (95% CI 1.13–1.86)], 24-h urinary sodium excretion [OR 1.12 (95% CI 1.05–1.20), per 1000 mg/day increase] and living in Bangladesh [OR 3.15 (95% CI 2.21–4.51)] and Sri Lanka [OR 5.25 (95% CI (3.47–7.96)] (versus in Pakistan) were significantly associated with increased odds of CKD (Table 3). After introducing clinical and comorbid variables in the model, these variables remained strongly associated with CKD. Moreover, diabetes [OR 1.88 (95% CI 1.49–2.37)], diuretic use [OR 1.55 (95% CI 1.11–2.18)] and SBP [OR 1.07 (95% CI 1.03–1.12), per 5 mmHg increase] were independently associated with CKD (Table 3).

**Table 3:** Factors associated with CKD among individuals with hypertension in rural communities in Bangladesh, Pakistan and Sri Lanka

Factors	Model 1 (*n* = 2306)		Model 2 (*n* = 2250)	
	OR (95% CI)	P-value	OR (95% CI)	P-value
Age	1.06 (1.05–1.07)	<0.001	1.05 (1.04–1.07)	<0.001
Marital status		0.003		0.002
Married	1.00		1.00	
Unmarried	1.45 (1.13–1.86)		1.49 (1.15–1.93)	
24-h urine sodium excretion (mg/day, per 1000 mg/day increase)	1.12 (1.05–1.20)	<0.001	1.13 (1.05–1.21)	<0.001
Country		<0.001		<0.001
Pakistan	1.00		1.00	
Bangladesh	3.15 (2.20–4.51)	<0.001	3.46 (2.32–5.17)	<0.001
Sri Lanka	5.25 (3.47–7.96)	<0.001	5.60 (3.52–8.93)	<0.001
Diabetes	–	–		<0.001
No	–	–	1.00	
Yes	–	–	1.88 (1.49–2.37)	
SBP (per 5** **mmHg increase)	–	–	1.07 (1.03–1.12)	<0.001
Diuretics use	–	–		0.011
No	–	–	1.00	
Yes	–	–	1.55 (1.11–2.18)	

Model 1: Logistic regression analysis, including age, gender, education, marital status, wealth index score, physical activities score, smoking, fruit consumption per week, 24-h urine sodium excretion and country.

Model 2: variables in Model 1 plus BMI, waist circumferences, diabetes, SBP, DBP, cardiovascular disease, HDL, LDL, triglyceride, diuretics use, ACEI or ARB use, CCB use and β-blocker use.

Variables with P <0.05 in both models were reported.

We also found significant interactions of country (P < 0.05) with age, marital status, SBP, LDL and 24-h sodium intake (Supplementary data, Table S6). Therefore we examined factors associated with CKD in each country (Table 4). The overall results were consistent, with the following exceptions: the positive association of age with CKD was less pronounced in Bangladesh than in Sri Lanka [Bangladesh versus Sri Lanka: ROR 0.94 (95% CI 0.91–0.96)] (Supplementary data, Table S6), but was not observed in Pakistan (Table 4) and 24-h sodium excretion was more equally associated with CKD in Pakistan and Sri Lanka (Supplementary data, Table S6), but not in Bangladesh (Table 4). Higher HDL was significantly associated with lower CKD risk only in Pakistan, higher LDL was associated with greater CKD risk only in Sri Lanka and diuretic use was not significant in all three countries (Table 4).

**Table 4:** Factors associated with CKD among individuals with hypertension stratified by rural communities in Bangladesh, Pakistan and Sri Lanka

Factors	Bangladesh (*n* = 823)		Pakistan (*n* = 661)		Sri Lanka (*n* = 766)	
	OR (95% CI)	P-value	OR (95% CI)	P-value	OR (95% CI)	P-value
Age	1.04 (1.02–1.06)	<0.001	1.00 (0.97–1.02)	0.69	1.11 (1.08–1.14)	<0.001
Marital status		0.99		0.15		0.004
Married	1.00		1.00		1.00	
Unmarried	1.00 (0.63–1.57)		1.54 (0.86–2.78)		1.97 (1.25–3.10)	
24-h urine sodium excretion (mg/day, per 1000 mg/day increase)	0.93 (0.83–1.04)	0.19	1.28 (1.09–1.50)	0.003	1.27 (1.11–1.45)	<0.001
Diabetes		<0.001		0.029		0.001
No	1.00		1.00		1.00	
Yes	2.13 (1.42–3.18)		1.89 (1.07–3.36)		1.94 (1.31–2.87 )	
SBP (per 5 mmHg increase)	1.08 (1.01–1.16)	0.021	1.18 (1.08–1.28)	<0.001	1.03 (0.96–1.11)	0.44
HDL	1.00 (0.99–1.02)	0.80	0.97 (0.95–0.99)	0.014	1.00 (0.98–1.03)	0.73
LDL (per 10 mg/dL increase)	0.97 (0.93–1.01)	0.10	0.95 (0.88–1.02)	0.18	1.07 (1.01–1.12)	0.021
Diuretics use		0.69		0.39		0.18
No	1.00		1.00		1.00	
Yes	1.16 (0.55–2.44)		1.77 (0.48–6.52)		1.37 (0.86–2.20)	

Variables in the model based on logistic regression analysis were age, gender, education, marital status, wealth index score, physical activities score, smoking, fruit consumption per week, 24-h urine sodium excretion, country, BMI, waist circumferences, diabetes, DBP, cardiovascular disease, HDL, LDL, triglyceride, diuretics use, ACEI or ARB use, CCB use and β-blocker use.

Sensitivity analysis showed that overall and country-specific CKD prevalence was lower using the original the CKD-EPI formula [19] than using the CKD-EPI Pakistan formula [26] in all countries (Table 2 and Supplementary data, Table S7). However, the relative magnitude of prevalence across countries remained unchanged and similar results were found for correlates of CKD using the CKD-EPI Pakistan formula (Supplementary data, Table S8). We also found that the association between 24-h urinary sodium excretion and CKD remained when using the Tanaka *et al*. [21] and Mage *et al*. [23] formulas (data not shown in tables).

## AWARENESS AND MANAGEMENT OF CKD

Of the 896 individuals with CKD, only 4.2% (95% CI 2.9%–5.6%) knew they had CKD, with 4.1% in Bangladesh, 2.6% in Pakistan and 4.7% in Sri Lanka. Moreover, only 31.0% (*n* = 278) controlled their BP to <140/90 mmHg and 16.3% (*n* = 146) to <130/80 mmHg. Furthermore, 21.5% (*n* = 193) were receiving statins and 33% (*n* = 191) with proteinuria were receiving an ACEI or ARB.

## DISCUSSION

Ours is the first study of its kind using a robust common protocol across rural communities in three South Asian countries: Bangladesh, Pakistan and Sri Lanka. We observed a high prevalence of CKD with striking cross-country variation. CKD prevalence was alarmingly high in Sri Lanka, affecting more than one in two individuals with hypertension, followed by one in three in Bangladesh and about one in six in Pakistan. Older age, being unmarried, higher 24-h urine sodium excretion, higher SBP, presence of diabetes, diuretic use and living in Sri Lanka and Bangladesh (versus in Pakistan) each were independently associated with greater odds of CKD. However, these conventional risk factors could not explain the cross-country variation in CKD prevalence, and especially the high prevalence in Sri Lanka. Among individuals with CKD, <5% knew their condition in each country and 69% had poor BP control.

Of note, a CKD prevalence of such a high magnitude in Sri Lanka affecting more than one in two hypertensive individuals has not been reported in opportunistic screening of high-risk individuals (including those with hypertension) elsewhere [27, 28]. The prevalence of CKD in Pakistan and Bangladesh is consistent with previous reports, albeit from the general population in urban areas of these countries [8, 29]. South Asia is experiencing a rapid epidemiological transition, and a substantial increase in BP levels and prevalence of diabetes has been reported from 1980 to 2008 [30, 31]. We observed that ∼27% of all participants in the three countries had diabetes, and this proportion was twice as high in Sri Lanka compared with Bangladesh or Pakistan. Also, more individuals used an ARB or ACEI in Sri Lanka (63.6%) than in Bangladesh (22.2%) and Pakistan (14.7%). However, the higher prevalence of CKD in Sri Lanka relative to the other two countries could not be explained by diabetes, ARB or ACEI use, SBP levels or other clinical risk factors because the OR associated with Sri Lanka remained statistically significant with either direct adjustment for these factors in the multivariate model or restriction of the analysis within a subgroup free of a certain risk factor. There are numerous reports of CKD of unknown etiology (CKDu), a significant contributor to the burden of CKD in rural Sri Lanka, although the prevalence of CKD reported in the literature (15.1–22.9%) is much lower than observed in our study [32]. The conventional definition of CKDu requires exclusion of high BP—an inclusion criteria for our study—although patients with CKD develop secondary hypertension and therefore this exclusion may be debatable [32]. The cause of CKDu has not yet been established and could be the interaction of multiple agents such as heavy metals, pesticides, native (Ayurvedic) medications or infections [32, 33]. Some selected clusters were near the North Central Province (NCP), considered one of the hotspots for CKDu [34]. However, we found high CKD prevalence in clusters far from the NCP as well, suggesting that other causes, including infections and herbal drug–related etiologies, might also underlie the high prevalence of CKD in Sri Lanka [35]. Further studies are necessary to unravel the reasons behind our findings.

We found that higher 24-h excretion of urinary sodium was associated with CKD, and the association was stronger in Pakistan and Sri Lanka. The 24-h excretion of urinary sodium is a reliable method of measuring dietary sodium intake, even in patients with reduced eGFR [36]. The mean sodium excretion was 4.9 g/day in our individuals with CKD, being substantially higher than the daily sodium intake recommended by the KDIGO guideline for CKD patients (<2 g/day) [24]. The levels were also higher than the mean global sodium intake of 3.9 g/day [37]. Controversy exists regarding the association between dietary sodium intake and the development or progression of CKD, with positive [38], inverse [39], U-shape [40] or no associations [41] reported in different studies. A recent randomized clinical trial found moderately improved renal outcomes in CKD patients with short-term sodium restriction [42]. There is limited evidence supporting an association between high sodium intake (>4.6 g/day) and adverse renal outcomes [43]. More studies are warranted to examine the association between dietary sodium intake and CKD.

Our findings concur with other studies showing that an increase in diuretics use is a poor prognostic indicator for kidney function decline and renal replacement therapy [44, 45]. However, this could potentially be a reflection of the BP treatment guidelines in CKD favoring diuretic-based regimens in advanced CKD [24].

Only 31% of all individuals with CKD in the three countries had adequately controlled BP to conventional targets of <140/90 mmHg. Recent evidence from the Systolic Blood Pressure Intervention (SPRINT) trial suggests strict BP control (SBP <120 mmHg) is more beneficial in preventing mortality in CKD patients [46]. Furthermore, the abysmally poor CKD awareness of <5% in our study was even lower than that (10%) for LMICs reported by the International Society of Nephrology’s Kidney Disease Data Center [29]. Our findings underscore the importance of intensive efforts to implement effective integrated programs for hypertension control and CKD screening and management in rural South Asia.

Our study has several limitations. First, the possibility of overestimation of CKD due to potential selection bias from missing data cannot be ruled out. However, this is least likely to influence our findings of high prevalence of CKD in Sri Lanka where missing data were minimal (Supplementary data, Figure S1). Additionally, the sampling was not representative of all rural areas in each country. However, CKD prevalence estimates were consistent in the clusters stratified by distance from a government clinic in each country, indicating that the burden is likely be uniformly high across rural areas (Supplementary data, Table S9). Second, the definition of CKD was based on a single measurement of serum creatinine and UACR as opposed to two measurements at least 3 months apart as recommended by the KDIGO [24] and therefore our results could overestimate the prevalence of CKD. However, a single measurement is common and acceptable for estimating the prevalence of CKD at the population level and it is unlikely to explain the cross-country variation observed in our study [47]. Third, the original CKD-EPI equation was used for individuals in Bangladesh and Sri Lanka, which has not been validated in the local populations. However, sensitivity analysis with the validated Pakistan-specific CKD-EPI equation in three countries further exaggerated the magnitude of CKD and its cross-country variation [26]. Fourth, the cross-sectional design of the study makes it impossible to infer a causal relationship between indicators of CKD and associated factors. Fifth, some possible determinants were not evaluated, such as access to health care, adverse environmental exposure and herbal medication use. Finally, we used a spot urine sample rather than a 24-h sample, which cannot provide a reliable sample of habitual sodium intake. However, a similar association was observed using the Tanka *et al*. [21] and Mage *et al*. [23] formulas, increasing confidence in the robustness of our results.

Major strengths of the study are the large sample from representative communities in chosen rural areas in multiple countries with door-to-door data collection, inclusion of near and far clusters in each country, uniform study design, a standardized protocol including standardized training of the research team and IDMS standardized serum creatinine measurements and collection of data during the same time period in all three countries, minimizing the potential of selection bias due to seasonal variation and enhancing cross-country comparability.

In conclusion, we observed an alarmingly high prevalence of CKD in adults with hypertension in rural Bangladesh, Pakistan and especially in Sri Lanka, with significant cross-country variation. The awareness of CKD was abysmally poor. Correlates of CKD were older age, being unmarried, higher sodium dietary intake, diabetes, elevated SBP and diuretic use. Our findings are of tremendous clinical and public health significance and call for urgent public health programs for CKD prevention, screening and management integrated with hypertension care services in rural South Asia. Further research to better understand the high prevalence of CKD is also warranted.

## Supplementary Material

gfy184_Supplementary_Data
