# Supplementary material for: Regional variation in chronic kidney disease and associated factors in hypertensive individuals in rural South Asia: findings from control of blood pressure and risk attenuation—Bangladesh, Pakistan and Sri Lanka
Source: Nephrol Dial Transplant. 2018 Jul 5;34(10):1723–30. doi: 10.1093/ndt/gfy184 (PMC6775474; doi:10.1093/ndt/gfy184)
Supplement: gfy184_Supplementary_Data [file gfy184_supplementary_data.doc]

**Supplementary Figure**


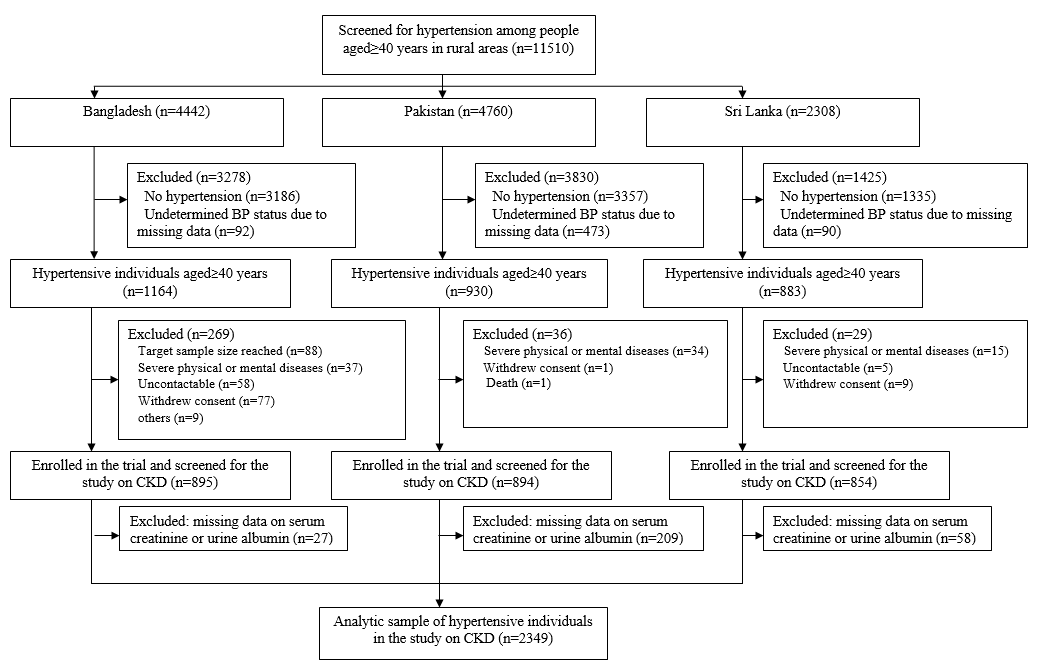


Figure S1: Study flow chart of hypertensive individuals included in the study on chronic kidney disease (CKD)

(30 clusters)

**Supplementary Tables**

Table S1: Comparison of baseline characteristics of hypertensive individuals included and excluded from the study on chronic kidney disease (CKD)

| Variables | Total N (N=2643) | Individuals included in the study (n=2349) |  | Individuals excluded from the study (n=294) | P value^a^ |
| --- | --- | --- | --- | --- | --- |
| Age, Mean(SD) |  | 58.8 (11.3) |  | 58.4 (12.8) | 0.61 |
| Male, n (%) |  | 835 (35.6) |  | 108 (36.7) | 0.69 |
| Marital status, n (%) |  |  |  |  | 0.012 |
| Unmarried |  | 620 (26.4) |  | 98 (33.3) |  |
| Married |  | 1729 (73.6) |  | 196 (66.7) |  |
| Education, n (%) |  |  |  |  | <0.001 |
| Formal |  | 1426 (60.7) |  | 131 (44.6) |  |
| Informal |  | 923 (39.3) |  | 163 (55.4) |  |
| IWI, n (%) | 2634 |  |  |  | <0.001 |
| ≥0 and <39.8 |  | 437 (18.7) |  | 87 (29.8) |  |
| ≥39.8 and <54.10 |  | 487 (20.8) |  | 46 (15.8) |  |
| ≥54.10 and <65.66 |  | 476 (20.3) |  | 47 (16.1) |  |
| ≥65.66 and <78.10 |  | 468 (20.0) |  | 61 (20.9) |  |
| ≥78.10 and <100 |  | 474 (20.2) |  | 51 (17.5) |  |
| Smoking, n (%) | 2642 | 242 (10.3) |  | 32 (10.9) | 0.76 |
| ≥1vegetable consumption per week, n (%) | 2639 | 2319 (98.9) |  | 288 (98.0) | 0.17 |
| ≥1 fruit consumption per week, n (%) | 2639 | 1460 (62.3) |  | 174 (59.2) | 0.31 |
| Physical activity score, MET-min/week, Median (IQR) | 2611 | 3360  (693,6759.6) |  | 1705 (198,6199.5) | <0.001 |
| BMI, Mean (SD) | 2588 | 24.7 (5.0) |  | 24.9 (5.9) | 0.59 |
| Waist circumference, Mean (SD) | 2633 | 88.2 (12.8) |  | 88.4 (14.9) | 0.78 |
| SBP, mmHg, Mean (SD) |  | 145.6 (21.7) |  | 146.8 (22.0) | 0.37 |
| DBP, mmHg, Mean (SD) |  | 88.3 (14.1) |  | 90.1 (15.4) | 0.051 |
| Diabetes, n (%) |  | 627 (26.7) |  | 43 (14.6) | <0.001 |
| CVD, n (%) | 2586 | 543 (23.7) |  | 42 (14.4) | <0.001 |
| Heart disease, n (%) | 2579 | 316 (13.8) |  | 26 (8.9) | 0.021 |
| Stroke, n (%) |  | 301 (12.8) |  | 23 (7.8) | 0.014 |
| ARB or ACEI use, n (%) |  | 800 (34.1) |  | 70 (23.8) | <0.001 |
| CCB, n (%) |  | 508 (21.6) |  | 46 (15.7) | 0.018 |
| Diuretics, n (%) |  | 248 (10.6) |  | 14 (4.8) | 0.002 |
| β-blocker, n (%) |  | 620 (26.4) |  | 48 (16.3) | <0.001 |
|  |  |  |  |  |  |

^a^ Continuous variables, two-sample t-test or Mann-Whitney U-test as appropriate; categorical variables, chi-square test

| N  (% (95%CI)) |  |  |  | Male |  |  |  |  | Female |  |
| --- | --- | --- | --- | --- | --- | --- | --- | --- | --- | --- |
|  |  | All n=835 | 40~50y n=183 | 50~65y n=364 | ≥65y n=288 |  | All n=1545 | 40~50y n=409 | 50~65y n=623 | ≥65y n=482 |
| eGFR<60 ml/min/1.73 m^2^ or UACR≥30 mg/g |  | 311  (37.3 (33.9,40.6) | 38 (20.8(14.6,26.9)) | 106  (29.1,(24.3,33.9)) | 167  (58.0 (52.1,63.9)) |  | 585  (38.6 (36.2,41.1) | 89  (21.8 (17.6,25.9)) | 207  (33.2 (29.5,37.0)) | 289  (60.0 (55.5,64.4) |
| eGFR<60 ml/min/1.73 m^2^ |  | 178 (21.4 (18.5,24.2) | 7  (3.8 (0.8,6.9)) | 48  (13.2 (9.6,16.8)) | 123  (42.7 (36.8,48.6)) |  | 326  (21.5 (19.4,23.6) | 13  (3.2 (1.4,5.0)) | 96  (15.4 (12.5,18.3)) | 217  (45.0(40.5,49.6)) |
| UACR≥30 mg/g |  | 197  (23.6 (20.7,26.5)) | 35  (19.1(13.2,25.1)) | 76  (20.9 (16.6,25.2)) | 86  (29.9 (24.4, 35.3)) |  | 376  (24.8 (22.6,27.0) | 82  (20.1 (16.1,24.1)) | 139  (22.3 (19.0,25.7)) | 155  (32.1(27.8,36.4)) |

**Table S2. Crude prevalence of chronic kidney disease (CKD) by age and gender among all individuals with hypertension (n=2349)**

eGFR, estimated glomerular filtration rate; UACR, urine albumin to creatinine ratio

**Table S3. Crude prevalence of chronic kidney disease (CKD) by age and gender among individuals with hypertension** in Bangladesh (n=868)

| N  (% (95%CI)) |  |  |  | Male |  |  |  |  | Female |  |
| --- | --- | --- | --- | --- | --- | --- | --- | --- | --- | --- |
|  |  | All n=314 | 40~50y n=64 | 50~65y n=147 | ≥65y n=103 |  | All n=554 | 40~50y n=214 | 50~65y n=229 | ≥65y n=111 |
| eGFR<60 ml/min/1.73 m2 or UACR≥30 mg/g |  | 129  (41.1 (35.5,46.7)) | 14 (21.9 (11.0,32.8)) | 54 (36.7,(28.6,44.9)) | 61  (59.2, (49.3,69.2)) |  | 187  (33.8 (29.7,37.8)) | 62 (29.0,(22.7,35.3)) | 72 (31.4,(25.2,37.7)) | 53  (47.8,(38.0,57.5) |
| eGFR<60 ml/min/1.73 m2 |  | 60  (19.1 (14.6,23.6)) | 2  (3.1 (0.0,8.2)) | 22  (15.0,(8.9,21.1)) | 36  (35.0,(25.3,44.7)) |  | 42  (7.6 (5.3,9.9)) | 6  (2.8 (0.4,5.3)) | 14  (6.1,(2.8,9.4)) | 22  (19.8 (12.0,27.7)) |
| UACR≥30 mg/g |  | 97  (30.9 (25.6,36.2)) | 14  (21.9 (11.0,32.8)) | 43 (29.3,(21.6,37.0)) | 40  (38.8 (28.9,48.7)) |  | 160  (28.9 (25.0,32.8)) | 59  (27.6 (21.4,33.8)) | 62 (27.1,(21.1,33.1)) | 39  (35.1 (25.8,44.5)) |

eGFR, estimated glomerular filtration rate; UACR, urine albumin to creatinine ratio

**Table S4. Crude prevalence of chronic kidney disease (CKD) by age and gender among individuals with hypertension in Pakistan (n=685**)

| N  (% (95%CI)) |  |  |  | Male |  |  |  |  | Female |  |
| --- | --- | --- | --- | --- | --- | --- | --- | --- | --- | --- |
|  |  | All n=269 | 40~50y n=85 | 50~65y n=111 | ≥65y n=73 |  | All n=416 | 40~50y n=130 | 50~65y n=174 | ≥65y n=112 |
| eGFR<60 ml/min/1.73 m2 or UACR≥30 mg/g |  | 49 (18.2 (13.4,23.0)) | 17 (20.0 (10.9,29.1)) | 13 (11.7,(5.3,18.1)) | 19  (26.0, (15.3,36.8)) |  | 67  (16.1 (12.5,19.8)) | 16  (12.3,(6.3,18.3)) | 28 (16.1,(10.3,21.8)) | 23  (20.5,(12.6,28.5) |
| eGFR<60 ml/min/1.73 m2 |  | 11  (4.1 (1.5,6.6)) | 1  (1.2 (0.0,4.1)) | 2  (1.8,(0,4.7)) | 8  (11.0,(3.1,18.8)) |  | 9  (2.2 (0.7,3.7)) | 2  (1.5 (0.0,4.0)) | 2  (1.2,(0.0,3.0)) | 5  (4.5 (0.2,8.7)) |
| UACR≥30 mg/g |  | 46  (17.1 (12.4,21.8)) | 17 (20.0 (10.9,29.1)) | 13 (11.7,(5.3,18.1)) | 16  (21.9 (11.7,32.1)) |  | 64  (15.4 (11.8,19.0)) | 16  (12.3 (6.3,18.3)) | 28 (16.1,(10.3,21.8)) | 20  (17.9 (10.3,25.4)) |

eGFR, estimated glomerular filtration rate; UACR, urine albumin to creatinine ratio

**Table S5. Crude prevalence of chronic kidney disease (CKD) by age and gender among individuals with hypertension in Sri Lanka (n=796**)

| N  (% (95%CI)) |  |  |  | Male |  |  |  |  | Female |  |
| --- | --- | --- | --- | --- | --- | --- | --- | --- | --- | --- |
|  |  | All n=252 | 40~50y n=34 | 50~65y n=106 | ≥65y n=112 |  | All n=544 | 40~50y n=65 | 50~65y n=220 | ≥65y n=259 |
| eGFR<60 ml/min/1.73 m2 or UACR≥30 mg/g |  | 133  (52.8 (46.4,59.1)) | 7 (20.6 (5.5,35.7)) | 39  (36.8,(27.1,46.4)) | 87  (77.7, (69.5,85.8)) |  | 331  (60.9 (56.7,65.0)) | 11 (16.9,(7.0,26.8)) | 107 (48.6,(41.8,55.5)) | 213 (82.2,(77.4,87.1) |
| eGFR<60 ml/min/1.73 m2 |  | 107 (42.5 (36.2,48.8)) | 4  (11.8 (0.0,24.0)) | 24  (22.6,(14.2,31.1)) | 79  (70.5,(61.7,79.4)) |  | 275  (50.6 (46.3,54.8)) | 5  (7.7 (0.5,14.9)) | 80 (36.4,(29.8,43.0)) | 190  (73.4 (67.8,78.9)) |
| UACR≥30 mg/g |  | 54 (21.4 (16.2,26.7)) | 4 (11.8 (0.0,24.0)) | 20  (18.9,(11.0,26.8)) | 30  (26.8 (18.1,35.4)) |  | 152  (27.9 (24.1,31.8)) | 7  (10.8 (2.5,19.1)) | 49 (22.3,(16.6,28.0)) | 96  (37.1 (31.0,43.1)) |

eGFR, estimated glomerular filtration rate; UACR, urine albumin to creatinine ratio

**Table S6. Ratio of odds ratio (ROR) between countries for variables that had significant interactions with country.**

| Variables | P value for interaction ^a^ |  | Bangladesh vs. Pakistan |  | Sri Lanka vs. Pakistan |  | Bangladesh vs. Sri Lanka |
| --- | --- | --- | --- | --- | --- | --- | --- |
|  |  |  | ROR (95% CI)(P value) |  | ROR (95% CI) (P value) |  | ROR (95% CI) (P value) |
| Age | <0.001 |  | 1.04 (1.01,1.07) (0.003) |  | 1.11 (1.08,1.15) (<0.001) |  | 0.94 (0.91,0.96) (<0.001) |
| Unmarried (VS married) | 0.024 |  | 0.62 (0.32,1.19) (0.15) |  | 1.37 (0.70,2.69) (0.36) |  | 0.45 (0.25,0.80) (0.007) |
| 24-hour urinary sodium secretion (mg/d, per 1000 mg/d increase) | <0.001 |  | 0.74 (0.62,0.89) (0.001) |  | 1.00 (0.82,1.21) (0.96) |  | 0.75 (0.63,0.88) (<0.001) |
| SBP (per 5mmHg increase) | 0.008 |  | 0.97 (0.91,1.04) (0.39) |  | 0.91 (0.85,0.97) (0.004) |  | 1.07 (1.01,1.13) (0.019) |
| LDL (per 10 mg/dL increase) | <0.001 |  | 1.04 (0.96,1.12) (0.33) |  | 1.15 (1.06,1.25) (<0.001) |  | 0.90 (0.85,0.96) (<0.001) |
|  |  |  |  |  |  |  |  |

Abbreviations: 95%CI, 95% confidence interval; SBP, systolic blood pressure; LDL, low density lipoprotein

^a^ P value for interaction with country in model 2 based on overall sample

**Table S7. Overall and country-specific prevalence of various stage of chronic kidney disease (CKD) among individuals with hypertension** (n=2349)

|  |  |  |  | CKD-EPI Pakistan equation ^a^ | | |
| --- | --- | --- | --- | --- | --- | --- |
| n (%, 95% CI) |  |  | Total  N=2349 | Bangladesh  N=868 | Pakistan N=685 | Sri Lanka  N=796 |
| CKD stage G3 ,A2 or worse |  |  | 1101 (46.9, (44.8,48.9)) | 348 (40.1 (36.8,43.4)) | 127 (18.5 (15.6,21.5)) | 626 (78.6 (75.7,81.6)) |
| CKD G3 or worse only |  |  | 766 (32.6,(30.7,34.5)) | 150 (17.3 (14.7,19.9)) | 34 (5.0 (3.3,6.7)) | 582 (73.1 (70.0,76.3)) |
| CKD G1 only |  |  | 767 (32.7 (30.7,34.6) | 295 (34.0 (30.8,37.2)) | 471 (68.8(65.2,72.3) | 1 (0.1 (0.0,0.4)) |
| CKD G2 only |  |  | 816 (34.7 (32.8,36.7)) | 423 (48.7 (45.4,52.1)) | 180 (26.3 (22.9,29.7) | 213 (26.8 (23.6,29.9)) |
| CKD G3a only |  |  | 495 (21.1 (19.4,22.7) | 97 (11.2 (9.0,13.3)) | 19 (2.8 (1.5,4.1)) | 379 (47.6 (44.1,51.2)) |
| CKD G3b only |  |  | 212 (9.0 (7.9,10.3)) | 37 (4.3 (2.9,5.7)) | 9 (1.3 (0.4,2.2)) | 166 (20.9 (18.0,23.7)) |
| CKD G4-5 only |  |  | 59 (2.5 (1.9,3.2)) | 16 (1.8 (0.9,2.8)) | 6 (0.9 (0.1,1.7)) | 37 (4.7 (3.1,6.2)) |

Abbreviations: CKD-EPI, the Chronic Kidney Disease-Epidemiology Collaboration; 95% CI,95% confidence interval
Stage G3 ,A2 or worse: eGFR<60 ml/min/1.73 m2 or UACR≥30 mg/g; G3 or worse only: eGFR<60 ml/min/1.73 m2 ; G1,eGFR≥90 ml/min/1.73 m2; G2,60 ml/min/1.73 m2≤ eGFR<90 ml/min/1.73 m2; G3, 30 ml/min/1.73 m2≤ eGFR<60 ml/min/1.73 m2; G3a,45 ml/min/1.73 m2≤ eGFR<60 ml/min/1.73 m2; G3b,30 ml/min/1.73 m2≤ eGFR<45 ml/min/1.73 m2; G4, 15 ml/min/1.73 m2≤ eGFR<30 ml/min/1.73 m2; G5, eGFR≤15 ml/min/1.73 m2

^a^ CKD-EPI Pakistan equation: 0.686 × original CKD-EPI^1.059^

**Table S8. Factors associated with chronic kidney disease (CKD) among individuals with hypertension in rural c**ommunities in Bangladesh, Pakistan, and Sri Lanka

|  |  | CKD-EPI Pakistan equation ^a^ | | | |
| --- | --- | --- | --- | --- | --- |
|  |  | Model1,n=2306 |  | Model 2,n=2250 | |
|  |  | OR (95%CI) | P value | OR (95%CI) | P value |
| Age |  | 1.06 (1.05,1.08) | <0.001 | 1.06 (1.05,1.07) | <0.001 |
| Marital status |  |  | 0.097 |  | 0.077 |
| Married |  | 1.00 |  | 1.00 |  |
| Unmarried |  | 1.25 (0.96,1.64) |  | 1.29 (0.98,1.70) |  |
| 24-hour urine sodium excretion (mg/d, per 1000 mg/d increase) |  | 1.08 (1.01,1.16) | 0.023 | 1.08 (1.00,1.16) | 0.040 |
| Country |  |  | <0.001 |  | <0.001 |
| Pakistan |  | 1.00 |  | 1.00 |  |
| Bangladesh |  | 3.34 (2.37,4.70) | <0.001 | 3.55 (2.40,5.23) | <0.001 |
| Sri Lanka |  | 13.28 (8.80,20.04) | <0.001 | 15.46 (9.68,24.72) | <0.001 |
| Diabetes |  | — | — |  | <0.001 |
| No |  | — | — | 1.00 |  |
| Yes |  | — | — | 1.82 (1.42,2.34) |  |
| SBP (per 5mmHg increase) |  | — | — | 1.09 (1.05,1.14) | <0.001 |
| Diuretics use |  | — | — |  | 0.083 |
| No |  | — | — | 1.00 |  |
| Yes |  | — | — | 1.41 (0.95,2.09) |  |
|  |  |  |  |  |  |

Abbreviations: CKD-EPI, the Chronic Kidney Disease-Epidemiology Collaboration; OR, odds ratio; 95% CI, 95% confidence interval; SBP, systolic blood pressure

Model 1: Logistic regression analysis. Age, gender, education, marital status, wealth index score, physical activities score, smoking, fruit consumption per week, 24-hour urine sodium excretion, and country.
Model 2: variables in model 1 plus body mass index (BMI), waist circumferences, diabetes, SBP, diastolic blood pressure (DBP), cardiovascular disease, high density lipoprotein (HDL), low density lipoprotein (LDL), triglyceride, diuretics use, angiotensin-converting enzyme inhibitor (ACEI) or angiotensin II receptor blocker (ARB) use, calcium channel blocker (CCB) use, and β-blocker use.

Variables with P<0.05 in both models were reported.

^a^ CKD-EPI Pakistan equation: 0.686 × original CKD-EPI^1.059^

**Table S9. Prevalence of chronic kidney disease (CKD) among individuals with hypertension stratified by distance of clusters ^a^** in each country

| Distance | Bangladesh | Pakistan | Sri Lanka |
| --- | --- | --- | --- |
| Near, n/N^b^ (%, 95% CI) | 193/524 (36.8 (32.6,41.1)) | 70/414 (16.9 (13.2,20.6)) | 261/477 (54.7 (50.2,59.3)) |
| Far , n/N^b^ (%, 95% CI) | 123/344 (35.8 (30.6,41.0)) | 46/271 (17.0 (12.3,21.6)) | 203/319 (63.6 (58.2,69.1)) |
|  |  |  |  |

^a^ the distance between a cluster and the government primary care clinic. Near clusters were defined as ≤2.5 km from government primary care clinic, and far cluster were defined as >2.5 km.

^b^ n was the number of hypertensive individuals with CKD, N was the total number of hypertensive individuals .
